# Fluoroquinolone treatment as a protective factor for 10-day mortality in *Streptococcus pneumoniae* bacteremia in cancer patients

**DOI:** 10.1038/s41598-021-81415-0

**Published:** 2021-02-12

**Authors:** Naihma Salum Fontana, Karim Yaqub Ibrahim, P. R. Bonazzi, F. Rossi, S. C. G. Almeida, F. M. Tengan, M. C. C. Brandileone, E. Abdala

**Affiliations:** 1 Instituto do Câncer do Estado de São Paulo da Faculdade de Medicina da Universidade de São Paulo, São Paulo, Brazil; 2 Departamento de Moléstias Infecciosas e Parasitarias da Faculdade de Medicina da Universidade de São Paulo, São Paulo, Brazil; 3 Divisão de Laboratório Central do Hospital das Clínicas da Universidade de São Paulo, São Paulo, Brazil; 4 Laboratório Nacional Para Meningites e Infecções Pneumocócicas do Instituto Adolfo Lutz, São Paulo, Brazil; 5 Rua Pandiá Calógeras, 445, Jardim Vergueiro, Sorocaba, São Paulo CEP 18030030 Brazil

**Keywords:** Cancer, Bacterial infection

## Abstract

To evaluate the prognostic factors in adult cancer patients with pneumococcal bacteremia, describe episode features and the phenotypic characteristics of the isolated strains. We evaluated the episodes in patients admitted to a cancer hospital between 2009 and 2015. The outcomes were defined as 48 h mortality and mortality within 10 days after the episode. The variables evaluated were: age, sex, ethnicity, ECOG, Karnofsky score, SOFA, cancer type, metastasis, chemotherapy, radiotherapy, neutropenia, previous antibiotic therapy, community or healthcare-acquired infection, comorbidities, smoking, pneumococcal vaccination, infection site, presence of fever, polymicrobial infection, antimicrobial susceptibility, serotype and treatment. 165 episodes were detected in 161 patients. The mean age was 61.3 years; solid tumors were the most prevalent (75%). 48 h and 10-day mortality were 21% (34/161) and 43% (70/161) respectively. The 48 h mortality- associated risk factors were SOFA and polymicrobial bacteremia; 10-day mortality-associated risk factors were fever, neutropenia, ECOG 3/4, SOFA and fluoroquinolones as a protective factor. Pneumococcal bacteremia presented high mortality in cancer patients, with prognosis related to intrinsic host factors and infection episodes features. Fluoroquinolone treatment, a protective factor in 10-day mortality, has potential use for IPDs and severe community-acquired pneumonia in cancer patients.

## Introduction

*Streptococcus pneumoniae* is an important causative agent of diseases around the world. Clinical manifestations range from mild presentations to invasive pneumococcal disease (IPD). IPDs are most prevalent in extremes of age—children under 2 years of age and adults over 65 years, as well as in patients with chronic pulmonary comorbidities, and immunocompromised hosts^1^. Cancer represents an important risk factor for IPD. In patients with lung cancer, the risk of IPDs is 13.41 times compared to the general population, and this risk is much higher for multiple myeloma^2^. IPDs cause high mortality in cancer patients compared to the general population, with reports of up to 40% within 7 days and 55% within 30 days after the first day of infection^3–6^. The need for further studies is shown by the high prevalence of pneumococcal bacteremia in the population with cancer, the high morbimortality related to them and the scarce world literature on epidemiology, etiology and bacteremia outcomes in these patients. Most of the available literature is restricted to those with hematological neoplasms, neutropenic and hematopoietic stem cell receptors. The objectives of this study were to evaluate prognostic factors in cancer patients in episodes of pneumococcal bacteremia and to describe the epidemiological, clinical and microbiological data, the phenotypic characteristics of the collected specimens (serotypes and antimicrobial resistance) and to correlate the *S. pneumoniae* serotypes with available vaccine coverage.

## Methods

### Study design and patients

A retrospective cohort study with inclusion of patients attended between January 2009 and July 2015 in the Institute of Cancer of the State of São Paulo (ICESP), Faculty of Medicine, University of São Paulo (FMUSP), with hematological malignancies and / or solid tumors, over 18 years of age, and who had at least one episode of *S. pneumoniae* bacteremia during the referred period. Bacteremia was defined as a clinical condition of infection with a blood culture positive for a microorganism. Each episode of bacteremia was considered as one case; other subsequent episodes in the same patient, during the period studied, were excluded from statistical analysis.

The Research Ethics Committee of the Faculty of Medicine of the University of São Paulo issued the opinion number 1,645,166 in which it states that the study in question is observational, retrospective and without intervention in the studied population and, therefore, without the possibility of ethical conflicts. Thus, this research was exempt from the application of terms of consent by our Ethics Committee. In this article, all methods have been carried out in accordance with the guidelines, relevant regulations and ethical principles for medical research guided by the Declaration of Helsinki.

### Microbiological analysis

*Streptococcus pneumoniae* was identified using standard methodology^7^ and the serotypes were identified by Quellung reaction using antisera from the Statens Serum Institute (Copenhagen, Denmark). Antimicrobial susceptibility testing was assessed via the disk diffusion method to 1 µg oxacillin (OXA) screening for susceptibility to penicillin (PEN), chloramphenicol (CLO), erythromycin (ERY), clindamycin (CLI), vancomycin (VAN), levofloxacin (LEV) sulfamethoxazole-trimethoprim (TMP/SMX), and tetracycline (TET) as recommended by the Clinical Laboratory Standards Institute guidelines^8^. Strains with OXA resistance (halo ≤ 19 mm) were analyzed for minimum inhibitory concentration (MIC) to PEN and ceftriaxone (CRO). Susceptibility to antimicrobial agents (susceptible, intermediate and resistant) was based on 2016 CLSI breakpoints according to clinical diagnosis^8^. Strains were considered susceptible to PEN for treatment of pneumococcal meningitis when MIC was ≤ 0.06 mg/L. For treatment of other pneumococcal infections, we considered MIC ≤ 2 mg/L. Regarding sensitivity to CRO, we considered MIC ≤ 0.5 mg/L for pneumococcal meningitis and MIC ≤ 1 mg/L for other pneumococcal infections^7,8^.

### Outcomes

The evaluated outcomes were 48-h mortality and 10-day mortality after the onset of episode of bacteremia.

### Definitions and variables

Neutropenia was defined as absolute neutrophil count ≤ 500 cells/mm^3^. Severe neutropenia was defined as neutrophils ≤ 100 cells/mm^3^, and prolonged neutropenia, such as that lasting 7 days.

The following variables were collected from patients’ medical records: gender, age, ethnicity, cancer diagnosis, Karnofsky, ECOG (scale of performance status), SOFA score, presence of metastases, current neutropenia, severe neutropenia, prolonged neutropenia. Chemotherapy, monoclonal antibodies, radiotherapy, corticotherapy, antibiotic therapy (ABT) in the past month (one month before IPD starts), comorbidities, smoking, and previous pneumococcal vaccine.

In addition to the demographic data, the epidemiological characterization of the episode was performed: community infection or infection related to health care (HAI—defined as manifested, clinically or microbiologically, after 48 h of hospital admission or if it occurred in an outpatient, who had undergone any invasive procedure or chemotherapy within 30 days prior to infection), site of infection, antimicrobial treatment, and polymicrobial bacteremia (blood culture positive for *S. pneumoniae* and another agent concomitantly in the same sample).

### Statistical analysis

All episodes were included for descriptive and statistical analysis of all variables. Mortality was described in 48 h and up to 10 days according to the characteristics evaluated and the association of characteristics with outcomes was verified using chi-square test, likelihood ratio test or Fisher's exact test and comparing the quantitative characteristics according to endpoints with use of Mann–Whitney test or Student's t-test. Odds Ratio (OR) of each variable was estimated with the respective 95% confidence intervals using bivariate logistic regression.

All variables with p < 0.10 in the bivariate tests were tested in the multiple logistic regression, and the variables of the final models were selected using the stepwise method with Backward selection with input and exit by 5%.

To perform analysis the software IBM-SPSS for Windows version 20.0 was used, and for tabulation of the data the software Microsoft Excel 2003 was used. The tests were performed with significance level of 5%.

### Ethics approval and consent to participate

All data were analyzed anonymously and confidentially, with approval by the Research Ethics Committee of Clínicas Hospital of the University of Sao Paulo and received approval by CONEP (National Ethics Commission), Brazil. It was a retrospective cohort study; thus, it was not possible to apply consent to participate.

## Results

In the study period, 165 episodes of *S. pneumoniae* bacteremia were identified in 161 patients. For the analysis, 4 patients who had two or more episodes (relapses) were excluded. Thus, 161 episodes in 161 patients were included in the statistical analysis.

The demographic and clinical characteristics of the individuals are described in Table 1. Data related to the episodes of infection are described in Table 2. Mortality in 48 h was 21% (34/161), and in 10 days 43% (70/161). Regarding onco-hematological tumors, there was 13% (5/40) ​​mortality in 48 h and 28% (11/40) in 10 days. Among solid tumors, 24% (29/121) in 48 h and 49% (59/121) in 10 days.

**Table 1:** Description of the demographic characteristics evaluated in 161 cancer patients with* Streptococcus pneumoniae* bacteremia in cancer patients.

Demographic Characteristic	Description, n (%)
**Sex**	
Female	60 (37.3)
Male	101 (62.7)
**Age (years)**	
mean ± SD	61.7 ± 11.9
median (min; max.)	62.1 (27.2; 91.1)
**Ethnicity**	
Asian	2 (1.2)
White	104 (64.6)
Black	27 (16.8)
Mixed	28 (17.4)
**Neoplasm, n (%)**	
**Solid tumors**	**121 (75.2%)**
Head and neck^a^	24 (14.9)
GTI	44 (27.3)
Respiratory tract	23 (14.3)
Liver/bile ductes	10 (6.2)
Breast	6 (3.7)
Pancreas	4 (2.5)
Female genitourinary	4 (2.5)
Bones/cartilage/SST	4 (2.5)
Male genitourinary	1 (0.6)
Timo/parotid	1 (0.6)
**Hematological neoplasms**	**40 (24.8%)**
Multiple myeloma	20 (12.4)
Non-Hodgkin's lymphoma	14 (8.7)
Hodgkin's lymphoma	4 (2.5)
Leukemias	2 (1.2)
**Comorbidities**	
Systemic arterial hypertension	75 (46.6)
Diabetes mellitus	33 (20.5)
Congestive heart failure	12 (7.5)
COPD	78 (48.4)
Smoking	88 (54.7)
Splenectomy	1 (0.6)
AIDS/HIV	8 (5)
Metastasis	90 (55.9)
**Karnofsky**	
90/100	62 (40.3)
70/80	62 (40.3)
< 70	30 (19.5)
**ECOG**	
0/1	86 (54.8)
2/3/4	71 (45.2)

*GTI* gastrointestinal tract (esophagus, stomach, small intestine, colon, anus and rectum), *SST *skin and soft tissue, *COPD* chronic obstructive pulmonary disease.

^a^Head and neck, means oral cavity, larynx and pharynx.

**Table 2:** Description of the characteristics evaluated in 161 episodes of bacteremia by *Streptococcus pneumoniae* in cancer patients.

Episode features	Description n/N (%)
Community-acquired infection	53 (32.9)
Healthcare-acquired infections (HAIs)	108 (67.1)
**Primary site of infection, n (%)**	
Lung	128 (79.5)
Abdomen	16 (9.9)
Bloodstream infection	7 (4.3)
Central nervous system	3 (1.9)
Oropharynx/sinuses	2 (1.2)
Skin and soft tissues	2 (1.2)
Biliary tract	2 (1.2)
Urinary tract	1 (0.6)
**SOFA (points)**	
Mean ± SD	5.32 ± 3.55
Median (min.; max.)	5 (0; 17)
Fever in the 48 h preceding the episode	97 (60.2)
Antibiotics past month	50/160 (31.3)
Radiotherapy past month	28 (17.4)
Chemotherapy past month	94 (58.4)*
Monoclonal antibodies past month	4 (2.5)
Corticotherapy past month	50 (31.1)
**Neutropenia**	
Neutropenia past month	43 (26.7)
Current neutropenia	38 (23.6)
Current severe neutropenia	34 (21.1)
Prolonged neutropenia past month	10 (6.2)
Febrile neutropenia	30 (18.6)
**Strain susceptibility**	
PEN susceptibility MIC ≤ 2	140/141 (99.29)
PEN susceptibility MIC ≤ 0.06	112/141 (79.43)
CRO susceptibility MIC ≤ 1	145/145 (100)
CHL susceptibility MIC ≤ 0.5	139/145 (95.86)
CLI susceptibility	142/155 (91.61)
CLO susceptibility	154/155 (99.35)
ERY susceptibility	136/155 (87.74)
LEV susceptibility	155/155 (100)
SXT susceptibility	87 /154 (56.49)
TET susceptibility	125/152 (82.23)
VAN susceptibility	155/155 (100)
Vaccine serotypes PCV13^a^	63/123 (51)
Vaccine serotypes PPV23^b^	91/123 (74)
Pneumococcal vaccine prior to the episode	4/9 (45)
Pneumococcal vaccine at any time	9/161 (5.6)
**Antimicrobial treatment**	
Penicilins^c^	90/160 (56.3)
Glycopeptide/linezolid	82/160 (51.3)
3rd and 4th generation cephalosporin	72/160 (45)
Fluoroquinolones^d^	29/160 (18.1)
CLI	7/160 (4.4)
SXT	1/160 (0.6)
Polymicrobial bacteremia	24 (14.9)
**Outcomes**	
48 h mortality	33 (20.5)
10-day mortality	54 (33.5)

*Data available in only 74 of 94 episodes that mentioned prior chemotherapy.

*MIC* minimum inhibitory concentration, *PEN* penicillin, *CRO* ceftriaxone, *CHL* chloramphenicol, *ERY* erythromycin, *CLI* clindamycin, *VAN* vancomycin, *LEV* levofloxacin, *SXT* trimethoprim-sulfamethoxazole, *TET* tetracycline.

^a^PCV vaccine 13, or pneumococcal conjugate vaccine 13.

^b^PPV 23 vaccine, or pneumococcal polysaccharide vaccine 23.

^c^Oxacillin, amoxicillin, piperacillin-tazobactam.

^d^Moxifloxacin, levofloxacin.

**Figure 1:**
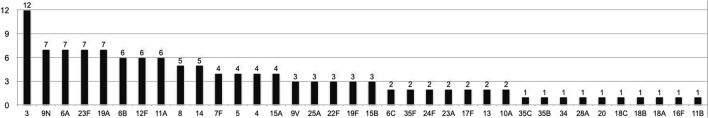
Distribution of serotypes in 123 strains on episodes of *Streptococcus pneumoniae* bacteremia in cancer patients.

Serotype information was available for 123 strains. The most prevalent serotype was 3, composing 12 episodes (10%), followed by 9 N, 6A, 23F, 19A (7/123; 6% each), 11A, 12F, 6B (6/123; 5% each) and serotypes 8 and 14 (5/123; 4% each). The other serotypes found are summarized in Fig. 1. In 64 episodes (52%), the serotype belonged to the pneumococcal conjugate 13-valent vaccine (PCV13); additionally, other 35 episodes (28%) were due to serotypes belonged to the strains of the pneumococcal polysaccharide 23-valent vaccine 23 (PPV23). Twenty-five (20%) serotypes were non-vaccine types.

Information on pneumococcal vaccination was obtained for only 45 patients; of these, 9 had received the PPV23 and only 4 had received the vaccine prior to the bacteremia episode. In the patients with previous pneumococcal vaccination, the serotypes found was 16F (non-vaccine type—NVT), 9N, 11A and 6B (vaccine-type).

Regarding the specific treatment of pneumococcal bacteremia, 12 patients (7%) did not receive any antimicrobials. Among the remaining 149, we detected 18 different antimicrobial association schemes. Forty-eight patients (30%) received some penicillin (PEN) associated with a glycopeptide (GLYCO), 26 (16%) received some cephalosporin (CEPH) in monotherapy, 13 (8%) received CEPH + PEN + GLYCO, 10 (6%) received CEPH plus fluoroquinolone (FQ), 10 (6%) received only PEN in monotherapy, 8 (5%) received CEPH + GLYCO, 7 (4%) received CEPH + PEN, and 7 (4%) received only some FQ. Other schemes: CEPH + FQ + GLYCO (5; 3%); CEPH + Clindamycin (CLI) (3; 2%); CEPH + PEN + GLYCO + CLI; CEPH + FQ + GLYCO; PEN + FQ and FQ + GLYCO corresponded to 2 patients each (1%). CEPH + PEN + CLI; GLYCO; PEN + GLYCO + TMP/SMX and FQ + CLI corresponded to 1 patient each (1%). Therefore, 56% (90/161) of the patients received some penicillin or a penicillin derivative. Half of the patients (82/161; 51%) received GLYCO or linezolid, 45% (72/161) received 3rd and 4th generation CEPH (ceftriaxone, ceftazidime or cefepime), 18% (29/161) received antibiotics from the FQ group (levofloxacin or moxifloxacin), 4% (7/161) received CLI and one patient (1%) received TMP/SMX as treatment. Fifty patients (50/161, 31%) received prophylaxis or antimicrobial treatment in the 30 days prior to the episode of pneumococcal bacteremia. Only antimicrobial regimen introduced specifically to treat the pneumococcal bacteremia in question (empirical or directive use) were included in the descriptive and statistical analysis.

The results of the bivariate analysis and multiple models of variables related to mortality in 48 h and 10 days are displayed in Tables 3 and 4, respectively.

**Table 3:** Factors potentially associated with 48 h mortality according to characteristics evaluated and results of bivariate and multivariate tests in 161 episodes of bacteremia caused by* Streptococcus pneumoniae* in cancer patients.

Variable	Bivariate analysis/multivariate analysis	
	OR (95% CI)	p value
**Sex**		0.183
Female	1	
Male	1.77 (0.76–4.11)	
Age (years)	0.98 (0.95–1.01)	0.122**
**Ethnicity**		0.061#
Asian	&	
White	0.71 (0.27–1.91)	
Black	0.52	
Mixed	1	
**Comorbidities**		
Systemic arterial hypertension	0.692 (0.317–1.51)	0.353
Diabetes mellitus	0.094 (0.012–0.71)	0.005
Congestive heart failure	&	0.128*
COPD	0.737 (0.34–1.60)	0.437
Smoking	0.628 (0.291–1.36)	0.234
Splenectomy	&	> 0.999*
AIDS/HIV	1.312 (0.252–6.82)	0.668*
Neoplasm		0.870#
**Solid tumors**		
Head and neck^a^	1	
GTI	0.67 (0.22–1.98)	
Respiratory tract	0.3 (0.07–1.32)	
Liver/biliary ducts	0.5 (0.09–2.93)	
Breasts	0.4 (0.04–4.02)	
Pancreas	0.67 (0.06–7.48)	
Female genitourinary	0.67	
Bones/cartilage/SST	0.67 (0.06–7.48)	
Male genitourinary	&	
Timo/parotid	&	
**Hematologic neoplasms**		
Multiple myeloma	0.22 (0.04–1.20)	
Non-Hodgkin's lymphoma	0.33 (0.06–1.86)	
Hodgkin's lymphoma	0.67 (0.06–7.48)	
Leukemias	&	
**Karnofsky**		0.116
90/100	1	
< 90	1.96 (0.84–4.59)	
**ECOG**		0.357
0/1/2	1	
3/4	1.49 (0.64–3.50)	
Presence of metastasis	1.496 (0.68–3.30)	0.315
Radiotherapy past month	1.73 (0.68–4.37)	0.244
Chemotherapy past month	1.56 (0.70–3.47)	0.279
Monoclonal antibodies past month	1.3 (0.13–12.94)	> 0.999*
Corticotherapy past month	1.14 (0.51–2.58)	0.751
Community-acquired infection	0.86 (0.38–1.97)	0.72
Health-care infection	1.16 (0.51–2.66)	0.72
**Primary site of infection**		0.216^#^
Lung	1	
Abdomen	0.45 (0.1–2.08)	
Bloodstream infection	&	
Central nervous system	&	
Oropharynx/facial sinuses	&	
SST	&	
Biliary tract	&	
Urinary tract	&	
Fever in the 48 h preceding the episode	0.64 (0.30–1.38)	0.25
**Neutropenia**		
Neutropenia past month	4.94 (2.19–11.14)	< 0.001
Current neutropenia	5.41 (2.37–12.39)	< 0.001
Severe neutropenia past month	4.54 (1.99–10.32)	< 0.001
Current severe neutropenia	4.78 (2.06–11.08)	< 0.001
Prolonged neutropenia past month	1.73 (0.42–7.08)	0.429*
Febrile neutropenia	2.87 (1.20–6.86)	0.015
Antibiotics last month	0.83 (0.35–1.95)	0.67
**SOFA**	1.4/1.37 (1.20–1.57)***	< 0.001£/ < 0.001***
**Polymicrobial bacteremia**	5.52 (2.19–13.94)/3.78 (1.26–11.32) ***	< 0.001*/0.018 ***
**Strain susceptibility**		
MIC PEN	0.57 (0.18—1.86)	0.771£
MIC CRO	0.87 (0.13–5.91)	0.856£
PEN susceptibility (MIC ≤ 2)	&	> 0.999*
CRO susceptibility (MIC ≤ 1)	&	&
CLI susceptibility	1.36 (0.27–6.50)	> 0.999*
CHL susceptibility	&	0.200*
ERY susceptibility	2.3 (0.50–10.55)	0.368*
LEV susceptibility	&	&
SXT susceptibility	1.99 (0.84–4.68))	0.113
TET susceptibility	2.32 (0.65–8.27)	0.184
VAN susceptibility	&	&
**Vaccination features**		
Vaccine serotype VCV 13^b^	2.64 (1.00–6.99)	&
Vaccine serotype VPV 23^c^	1.32 (0.45–3.9)	0.046
Pneumococcal vaccine before the episode	&	0.618
Pneumococcal vaccine in any time	&	> 0.999*
Antimicrobial treatment (ABT)		
Instituted in the first 24 h	0.47 (0.162–1.36)	0.212*
3rd and 4th generation cephalosporin	0.38 (0.164–0.88)	0.022
Penicillin^d^	0.92 (0.424–1.98)	0.825
Fluoroquinolones^e^	0.11 (0.014–0.85)	0.012
Glycopeptide/linezolid	0.75 (0.346–1.61)	0.455
SMX/TMP	&	> 0.999*
Clindamycin	1.57 (2.19–13.94)	0.634*

Chi-square test; *Fisher exact test; ^#^Likelihood ratio test; **T-Student Test; ^£^Mann–Whitney test; ^&^Cannot estimate; ***Multiple logistic regression.

*COPD* chronic obstructive pulmonary disease, *GTI* gastrointestinal tract (esophagus, stomach, small intestine, colon, anus and rectum), *SST* skin and soft tissue, *PEN* peniciin, *CRO* ceftriaxone, *CLO* chloramphenicol, *ERY* erythromycin, *CLI* clindamycin, *VAN* vancomycin, *LEV* levofloxacin, *SXT* sulfamethoxazole- trimethoprim, *TET* tetracycline.

^a^Head and neck, oral cavity, larynx and pharynx.

^b^Vaccine VCV 13. or pneumococcal conjugate vaccine 13.

^c^Vaccine VPV 23. or pneumococcal polysaccharide vaccine 23.

^d^Oxacillin, amoxicillin, piperacillin-tazobactam.

^e^Moxifloxacin, levofloxacin. *MIC* minimum inhibitory concentration.

**Table 4:** Factors potentially associated with 10-day mortality according to characteristics evaluated and results of bivariate and multivariate tests in 161 episodes of bacteremia caused by* Streptococcus pneumoniae* in cancer patients.

Variable	Bivariate analysis/multivariate analysis	
	OR (95% CI)	*p* value
**Sex**		0.034
Female	1	
Male	2.155 (1.05–4.42)	
Age (years)	1.007 (0.98–1.04)	0.643**
**Ethnicity**		0.159^#^
Asian	&	
White	1.118 (0.46–2.72)	
Black	0.739 (0.23–2.38)	
Mixed	1	
**Comorbidities**		
Systemic arterial hypertension	0.785 (0.41–1.52)	0.471
Diabetes mellitus	0.37 (0.14–1.52)	0.036
Congestive heart failure	0.373 (0.08–1.77)	0.340*
COPD	0.878 (0.46–1.69)	0.698
Smoking	0.754 (0.39–1.45)	0.399
Splenectomy	&	> 0.999*
AIDS/HIV	1.2 (0.28–5.22)	> 0.999*
Neoplasm		0.056#
**Solid tumors**		
Head and neck^a^	1	
GTI	1.079 (0.4–2.93)	
Respiratory tract	0.517 (0.16–1.71)	
Liver/bile ducts	0.295 (0.05–1.69)	
Breasts	0.591 (0.09–3.86)	
Pancreas	1.182 (0.14–9.83)	
Female genitourinary	0.394 (0.04 -4.35)	
Bones/cartilage/SST	0.394 (0.04–4.35)	
Male genitourinary	&	
Timo/parotid	&	
Hematologic neoplasms		
Multiple myeloma	0.131 (0.03–0.70)	
Non-Hodgkin's lymphoma	0.197 (0.04–1.08)	
Hodgkin's lymphoma	0.394 (0.04–0.82)	
Leukemias	&	
**Karnofsky**		0.053
90/100	1	
< 90	2.014 (0.98–4.12)	
**ECOG**		0.032
0/1/2	1	0.937
3/4	2.25 (1.06–4.77)/5.93 (2.04–17.22) ***	0.937/0.001***
Presence of metastases	2.218 (1.11–4.41)	0.022
Radiotherapy past month	1.354 (0.58–3.14)	0.479
Chemotherapy past month	0.941 (0.49–1.83)	0.858
Monoclonal antibodies past month	6.235 (0.63–61.43)	0.110*
Corticotherapy past month	1.331 (0.66–2.67)	0.421
Community-acquired infection	1.029 (0.51–2.06)	0.72
Health-care infection	0.972 (0.49–1.95)	0.72
**Primary site infection**		0.018^#^
Lung	1	
Abdomen	0.372 (0.10–1.37)	
Bloodstream infection	&	
Central nervous system	&	
Oropharynx/facial sinuses	&	
SST	1.612 (0.1–26.37)	
Biliary ducts	&	
Urinary tract	&	
Fever in the 48 h preceding the episode	0.42 (0.21–0.82)/0.351 (0.14–0.88)***	0.01/(0.026)***
**Neutropenia**		
Neutropenia past month	3.227 (1.56–6.67)	0.001
Current neutropenia	3.91 (1.83–8.36)/4.006 (1.38–11.65)***	< 0.001/0.011***
Severe neutropenia past month	3.369 (1.59–7.16)	0.001
Current severe neutropenia	3.908 (1.78–8.59)	< 0.001
Prolonged neutropenia past month	1.347 (0.36–4.99)	0.733*
Febrile neutropenia 48 h preceding the episode	2.359 (1.05–5.29)	0.034
Antibiotics last month	0.81 (0.40–1.67)	0.571
SOFA	1.4 (1.24–1.60)/1.407 (1.2–1.66)***	< 0.001£/ < 0.001***
Strain susceptibility	&	0.335*
MIC PEN	1.16 (0.62–2.19)	0.904£
MIC CRO	1.31 (0.27–6.23)	0.369£
PEN susceptibility (MIC ≤ 2)	&	0.333*
CRO susceptibility (MIC ≤ 1)	&	&
CLI susceptibility	1.089 (0.32–3.73)	> 0.999*
CHL susceptibility	&	0.329*
ERY susceptibility	1.98 (0.62–6.31)	0.241
LEV susceptibility	&	&
SXT susceptibility	1.345 (0.68–2.68)	0.398
TET susceptibility	2.017 (0.76–5.35)	0.153
VAN susceptibility	&	&
**Vaccination**		
Vaccine serotype VCV 13^b^	2.72 (1.21–6.14)	0.014
Vaccine serotype VPV 23^c^	0.915 (0.38–2.2)	0.842
Pneumococcal vaccine before the episode	&	0.539*
Pneumococcal vaccine in any time	&	&
Antimicrobial treatment		
Instituted in the first 24 h	0.359 (0.13–0.97)	0.038
3rd and 4th generation cephalosporin	0.331 (0.16–0.67)	0.002
Penicilins^d^	1.708 (0.87–63.36)	0.119
Fluoroquinolones^e^	0.053 (0.01–0.40)/0.083 (0.01–0.71)***	< 0.001/0.023***
Glycopeptide/linezolid	1.454 (0.75–2.81)	0.266
SMX/TMP	&	> 0.999*
Clindamycin	0.777 (0.15–4.14)	> 0.999*
Polymicrobial bacteremia	3.395 (1.39–8.28)	0.005

Chi-square test; *Fisher exact test; ^#^likelihood ratio test; **T-Student Test; ^£^Mann–Whitney Test; ^&^Cannot estimate; ***Multiple logistic regression.

*COPD* chronic obstructive pulmonary disease, *GTI* gastrointestinal tract (esophagus, stomach, small intestine, colon, anus and rectum), *SST* skin and soft tissue, *PEN* penicillin, *CRO* ceftriaxone, *CLO* chloramphenicol, *ERY* erythromycin, *CLI* clindamycin, *VAN* vancomycin, *LEV* levofloxacin, *SXT* trimethoprim-: sulfamethoxazole, *TET* tetracycline.

^a^Head and neck, oral cavity, larynx and pharynx.

^b^Vaccine VCV 13, or pneumococcal conjugate vaccine 13.

^c^Vaccine VPV 23. or pneumococcal polysaccharide vaccine 23.

^d^Oxacillin, amoxicillin, piperacillin-tazobactam.

^e^Moxifloxacin, levofloxacin.*MIC* Minimum inhibitory concentration.

Table 3 shows that, together, only SOFA and polymicrobial bacteremia were statistically associated with mortality in 48 h (p < 0.05), the increase of one unit in SOFA resulted in a 37% increase in the chance of mortality in 48 h and the chance of mortality in 48 h in patients with polymicrobial bacteremia was 3.78 times the chance of in patients without polymicrobial bacteremia.

Table 4 shows that, together, patients who had fever within the 48 h prior to the data of positive blood culture had a 65% lower chance of mortality within 10 days. Patients with current neutropenia had a chance of mortality within 10 days 4.01 times higher than the patients without current neutropenia. Patients with ECOG 3/4 presented 5.93 times the chance of mortality in up to 10 days compared to patients with ECOG 0/1/2. The increase of one unit in SOFA resulted in an increase of 59% chance of mortality up to 10 days. The chance of mortality up to 10 days in patients who used quinolones was 92% less.

## Discussion

Our sample consisted of 161 patients, 101 men (62.7%), with a mean age of 61.3 years. In fact, male gender and age over 65 years are risk factors reported for IPD, including in recurrent episodes^9^.

The most prevalent primary site of infection was the lung (128/161, 79.5%), followed by the abdomen (16/161, 9.9%). Pneumonia with lung as the primary site of infection was the most common clinical diagnosis among IPD in cancer patients, including pneumococcal bacteremia^10,11^.

In the present study, the four patients who presented two or more episodes had multiple myeloma (MM). The strong association of recurrent IPD and MM has been reported previously, and can be explained by intrinsic defects in humoral immunity and complement system, with opsonization dysfunction, an essential process in the granulocytic interaction with pneumococcus^2^.

The independent factors associated with mortality in 48 h in the multivariate analysis were the presence of polymicrobial bacteremia (PB) and SOFA. The 10-day mortality risk factors were current neutropenia, ECOG 3 or 4 and higher SOFA. The 10-day mortality protective factors were treatment with fluoroquinolones (FQ) and presence of fever.

Polymicrobial bacteremia has been associated with worse prognosis and unfavorable outcomes in several previous studies. In cancer patients, polymicrobial etiology significantly increases the risk of 30-day mortality and is associated with a higher propensity for severe sepsis and septic shock. This is an independent factor for worsening overall survival^12,13^.

The SOFA score is a condition that reflects organic failure in the context of sepsis^14^, being a consolidated predictor of prognosis in patients with cancer and independent factor for mortality. Each point earned in SOFA increased the chance of death by 17% during hospitalization in cancer patients in a previous study which 86% patients had solid tumors^13^. In the same study, ECOG was an independent predictor of in-hospital mortality when the value was 2, 3 or 4, which is consistent with other studies^15,16^ and with our finding in this present study, where ECOG 3 or 4 was a predictor of mortality in 10 days.

The prevalence of neutropenia in this study was 23.6%, consistent with other studies of pneumococcal bacteremia in oncology,18% to 26%^17^, and was a risk factor for 10-day mortality in the multivariate analysis. Numerous studies point to the presence of neutropenia as an independent and significant risk factor for mortality due to infection in cancer patients, mostly when dealing with pneumococcal bacteremia^4,18^.

Among the findings, treatment with FQ was a significant protective factor for 10-day mortality in the multivariate analysis, with a 92% lower chance of death. Previous studies have already shown consistent evidence about the effectiveness of the FQ in community-acquired pneumonia (CAP): a meta-analysis suggested that moxifloxacin alone had a higher pathogen eradication rate than a β-lactam (BL)-based combination therapy^19^, and also others studies showed that the use of FQ was associated with higher treatment success and better clinical outcomes compared to the established combination therapy of β-lactam antibiotic and a macrolide (ML) or without ML in general population^20^.

When bacteremic pneumonia in cancer patients arises, a population cohort showed that treatment with FQ or BL plus ML was a protective factor for 30-day mortality compared to BL monotherapy^5^. Similarly, in a retrospective chart review FQ (moxifloxacin) versus combination therapy in patients with severe CAP in intensive care unit (ICU) showed no difference in 30-day survival.

Thus, our findings are unprecedented and promising in favor of the use in monotherapy of FQ in IPD, which include pneumococcal bacteremia, bacteremic pneumonias and severe CAP.

The presence of fever in the 48 h before the onset of the episode was also a protective factor for 10-day mortality. Fever probably served as an alert, which led the patient to seek care earlier and, consequently, to receive antimicrobial treatment earlier, which is a decisive independent factor in sepsis-related mortality^14^.

Regarding antimicrobial susceptibility, our study presented a better susceptibility profile than other similar studies. Of all 141 strains tested, susceptibility to PEN was 79% (MIC ≤ 0.06 µg/mL) and 99% (MIC ≤ 2 µg/mL) which is higher than reported in the literature (74%, MIC ≤ 0.06 ug/mL^21^), including among cancer patients (78% MIC ≤ 0.1 ug/mL^22^ and 86% MIC ≤ 2 ug/mL^23^. In our study, no strains with high resistance to PEN were identified, and the MIC range was between 0.006 ug/mL and 4 ug/mL (median 0.016 ug/mL).

Of the 145 strains tested, the susceptibility to CRO was 100% (MIC ≤ 1 µg/mL, 4%? and MIC ≤ 0.5 µg/mL 96%). The median MIC was 0.016 µg/mL, with a range between 0.001 and 1 µg/mL. n other studies, the susceptibility (MIC ≤ 1 ug/mL) varied from 98%^23^ to 99%^21^.

Overall, pneumococcal susceptibility to other antimicrobials remained similar to those found in the literature, except small decrease of susceptibility to CLI and ERY^21,23^. It was not possible to statistically estimate the protective effect of pneumococcal vaccination due to the small number of patients known to be vaccinated (n = 9).

Among the 123 strains serotyped, 64 (52%) corresponded to those found in the PCV13 vaccine, and other 35 (28%) to the PPV23. 25 serotypes (20%) belonged to nonvaccine types, assuming that, hypothetically, 48 h and 10-day mortality would be potentially preventable through vaccination in 80% of the episodes of pneumococcal bacteremia.

The most prevalent serotypes found were serotype 3 (9.76%), followed by 19A, 23F, 6A, 9 N (5,69%) and 11A, 12F, 6B (4,88%). Of these, 3, 9 N and 6A are highly virulent, along with serotypes 19F and 6B. In particular, serotype 3 is described as being strongly encapsulated when compared to other serotypes^24,25^, which gives it a high virulence. In addition, types 19A and 23F have a high relative risk previously reported^24^.

In the present study, 48 h and 10-day mortality were, respectively, 20.5% and 33.5%. In similar studies, mortality in 48 h averaged 15% with a median of 13%^18,26–28^, and mortality at 30 days averaged 23.08%, with a median of 21.26%^2,18,26,27,29,30^. In previous multi-center studies^21,31^, there was a higher mortality rate from IPDs in general in Brazil and Latin America compared to other countries, which may explain, in part, the high mortality in this study. In addition, other factors possibly implicated would be infection with highly virulent serotypes^23,24^, especially serotype 3, the higher incidence of polymicrobial bacteremia, and the high incidence of neutropenia^12^.

The limitations of our study were its retrospective design, and the fact that it is a single center study with data obtained from medical records, which may have limited information available. There was no stratification of mortality by age and there was a shortage of vaccination history coverage information to estimate its protection role.

On the other hand, the study presents some aspects that make it unprecedented. Our case series was extensive (n = 161), and it is the only Brazilian study, so far, among the studies that evaluated prognostic factors in pneumococcal bacteremia in adult cancer patients. Due to the wide range of variables included, we could conclude several prognostic factors, confirm some previously known ones and include new hypotheses, such as the likely protective effect of FQ in the treatment of bacteremic pneumococcal pneumonia and severe CAP in cancer patients. This population is rarely targeted by clinical trials. Serotyping was performed in most cases (124/161), which provided important information about the prevalence, in addition to estimating the protective effect of the vaccine.

In conclusion, our study corroborated the high mortality associated with pneumococcal bacteremia in cancer patients. Factors associated with a worse prognosis were those intrinsically related to the host and to the episode itself. Despite the observation of high mortality rates in this study, the resistance rate to penicillin was lower than what previously was described in published series. The vast majority of isolated *S. pneumoniae* strains are included in the available vaccines, indicating the need for investment and optimization of vaccine focused prevention in cancer patients. FQ treatment as a protective factor in 10-day mortality shows its potential use for IPDs and severe CAP in cancer patients. Prospective studies should be conducted to confirm this finding in the future.

## Data Availability

The datasets used and/or analyzed during the current study are available from the corresponding author on reasonable request.

## Abbreviations

MIC: Minimal inhibitory concentration
ECOG: Eastern Cooperative Oncology Group
SOFA: Sequential Organ Failure Assessment score
