# Author Correction: Fluoroquinolone treatment as a protective factor for 10-day mortality in *Streptococcus pneumoniae* bacteremia in cancer patients

**DOI:** 10.1038/s41598-021-96034-y

**Published:** 2021-08-16

**Authors:** Naihma Salum Fontana, Karim Yaqub Ibrahim, P. R. Bonazzi, F. Rossi, S. C. G. Almeida, F. M. Tengan, M. C. C. Brandileone, E. Abdala

**Affiliations:** 1 Instituto do Câncer do Estado de São Paulo da Faculdade de Medicina da, Universidade de São Paulo, São Paulo, Brazil; 2 Departamento de Moléstias Infecciosas e Parasitarias da Faculdade de Medicina da, Universidade de São Paulo, São Paulo, Brazil; 3 Divisão de Laboratório Central do Hospital das Clínicas da, Universidade de São Paulo, São Paulo, Brazil; 4 Laboratório Nacional Para Meningites e Infecções Pneumocócicas do, Instituto Adolfo Lutz, São Paulo, Brazil; 5 Rua Pandiá Calógeras, 445, Jardim Vergueiro, Sorocaba, São Paulo, CEP 18030030 Brazil

Correction to: *Scientific Reports* 10.1038/s41598-021-81415-0, published online 12 February 2021

The original version of this Article contained an error in the spelling of the author Karim Yaqub Ibrahim which was incorrectly given as K. I. Ibrahim.

As a result, the Author Contributions section now reads:

“N.S.F. assembled the data and drafted the manuscript. F.R., M.C.C.B. and S.C.G.A. assisted in MIC and serotype determination; F.M.T., P.R.B. and K.Y.I. assisted with the draft of manuscript; and E.A. designed, supervised, and assessed the study and drafted the manuscript. All authors have read, contributed, and approved the final manuscript.”

The original Article has been corrected.

